# Enhanced Biocide Mitigation of Field Biofilm Consortia by a Mixture of D-Amino Acids

**DOI:** 10.3389/fmicb.2016.00896

**Published:** 2016-06-13

**Authors:** Yingchao Li, Ru Jia, Hussain H. Al-Mahamedh, Dake Xu, Tingyue Gu

**Affiliations:** ^1^College of Mechanical Engineering and Applied Electronics Technology, Beijing University of TechnologyBeijing, China; ^2^Department of Chemical and Biomolecular Engineering, Institute for Corrosion and Multiphase Technology, Ohio UniversityAthens, OH, USA; ^3^Saudi Basic Industries CorporationJubail, Saudi Arabia; ^4^Institute of Metal Research, Chinese Academy of SciencesShenyang, China

**Keywords:** biocide, biocide enhancer, D-amino acid, biofilm consortium, microbiologically influenced corrosion

## Abstract

Microbiologically influenced corrosion (MIC) is a major problem in the oil and gas industry as well as in many other industries. Current treatment methods rely mostly on pigging and biocide dosing. Biocide resistance is a growing concern. Thus, it is desirable to use biocide enhancers to improve the efficacy of existing biocides. D-Amino acids are naturally occurring. Our previous work demonstrated that some D-amino acids are biocide enhancers. Under a biocide stress of 50 ppm (w/w) hydroxymethyl phosphonium sulfate (THPS) biocide, 1 ppm D-tyrosine and 100 ppm D-methionine used separately successfully mitigated the *Desulfovibrio vulgaris* biofilm on carbon steel coupons. The data reported in this work revealed that 50 ppm of an equimolar mixture of D-methionine, D-tyrosine, D-leucine, and D-tryptophan greatly enhanced 50 ppm THPS biocide treatment of two recalcitrant biofilm consortia containing sulfate reducing bacteria (SRB), nitrate reducing bacteria (NRB), and fermentative bacteria, etc., from oil-field operations. The data also indicated that individual D-amino acids were inadequate for the biofilm consortia.

## Introduction

Microbiologically influenced corrosion (MIC) was first reported over 100 years ago (Gaines, 1910). It has become a major problem in the oil and gas industry in recent years because water injection is practiced more frequently than ever to increase well pressure. In this process, microbes and nutrients are introduced into the reservoirs and they subsequently show up in the pipelines, leading to MIC. MIC was suspected as a major contributing factor in the pipeline leak caused by a 0.25′′ × 0.5′′ hole at the bottom of a pipe in the Trans-Alaska Pipeline in 2006 (Jacobson, 2007). In a separate reported case, an 8′′ diameter pipeline carrying oil and produced water failed in only 8 months due to MIC (Bhat et al., 2011). A recent pipeline failure case was published suggesting that MIC was likely the culprit in the leak of a 24′′ CO_2_ gathering line 2 years after commissioning, after ruling out other corrosion mechanisms (Hinkson et al., 2013). Skovhus and Eckert (2014) discussed several MIC cases and pointed out that MIC is becoming more prevalent nowadays due to aging equipment and increased awareness.

In the MIC caused by biofilms of sulfate reducing bacteria (SRB), elemental iron (Fe^0^) in carbon steel serves as an electron donor. Xu and Gu (2014) designed an SRB starvation experiment to support this hypothesis. They found that under carbon source starvation, MIC pitting accelerated despite the fact that the biofilm lost some cell mass due to the starvation. SRB switched to Fe^0^ as electron donor for energy production to help their survival. Rajala et al. (2015) suggested that the presence of carbon steel benefited the microbial community in a nutrient-deficient anaerobic environment. In this kind of MIC, cross-cell wall electron transport is required for SRB to utilize extracellular electrons released by Fe^0^ oxidation in their cytoplasm for sulfate reduction, meaning that the biofilm must be electrogenic (Gu, 2012). Recent studies further suggested that electron transfer is a bottleneck in SRB MIC against carbon steel and 304 stainless steel (Li et al., 2015; Zhang et al., 2015). MIC by acid-producing bacteria (APB) belongs to a different type of MIC because the oxidant (proton) is reduced outside the cells on the steel surface rather than in the cytoplasm (Gu, 2012). Underneath an APB biofilm, the local pH is much more acidic than in the bulk-fluid pH, leading to organic acid attacks. In both types of MIC discussed above, biofilms are responsible. Thus, biofilm mitigation is a key to MIC mitigation.

In the field, microbes form synergistic biofilm consortia. It is well known that biofilm cells (sessile cells) are far more recalcitrant than planktonic cells. Through several different mechanisms, a biofilm protects inner sessile cells from harmful factors. In one way, a biofilm can slow down the diffusion of antimicrobial agents (Mah and O’Toole, 2001; Stewart and Costerton, 2001). Furthermore, it can slow down the metabolic rate to reduce the intake of antimicrobial agents. Tuomanen et al. (1986) found that the increased resistance to antibacterial agents was accompanied by a low growth rate or no growth. Biofilms also preserve persister cells when they are under attack. These persister cells quickly rebound when the environment becomes less hostile (Lewis, 2001). Sessile cells in biofilms can also use eﬄux pumps to prevent antimicrobial agents from entering the cells. They can upregulate resistant genes to break down antimicrobial agents (Lewis, 2001). These anti-microbial mechanisms make the mitigation of biofilm consortia difficult. It is said that as a rule of thumb, a ten times or higher biocide concentration may be required to treat biofilms compared with that needed for the treatment of planktonic cells (Nickel et al., 1985; Videla, 1996; Vance and Thrasher, 2005).

Pigging and biocides are two primary ways to mitigate problematic biofilms (Videla, 2002). Biocide applications can be performed during pigging by placing a biocide “plug” in a pipeline between two pigs. However, some pipelines are not piggable due to complicated elbows and other devices (Tiratsoo, 2013). Since pigging cannot completely remove biofilms, it is likely that the residual sessile cells can bounce back quickly. A biocide is needed to delay the recovery of biofilms. Tetrakis hydroxymethyl phosphonium sulfate (THPS) is one of the widely used biocides in the oil and gas industry because it is biodegradable and effective against a broad spectrum of microorganisms. THPS is designated by the US Environmental Protection Agency (EPA) as a green chemical (United States Environmental Protection Agency, 1997). It disrupts the disulphide bond in proteins and enzymes in microbes (Denyer, 1995; Russell, 2002; Ballantyne and Jordan, 2004; Greene et al., 2006). In field operations, repeated use of the same biocide will inevitably cause dosage escalation because of the selective promotion of resistant microbes over time. In some field operations, THPS dosage is so high such that the sulfate introduced by THPS precipitates with barium in the drilling fluid causing problematic scale formation downhole. High doses of biocides also raise environmental concerns in addition to increased cost. More effective uses of existing biocides are highly desirable.

Despite continued research in new biocides, it is unlikely that a blockbuster biocide will replace THPS or glutaraldehyde (another popular green biocide for large-scale applications) in oilfield applications any time soon. Thus, it is desirable to use biocide enhancers to make existing biocides such as THPS more effective. D-Amino acids were reported to enhance the efficacy of THPS in the mitigation of the *Desulfovibrio vulgaris* biofilm (Xu et al., 2012a,b, 2014). Most recently, Jia et al. (2016) demonstrated that D-amino acids enhanced two other biocides, namely alkyldimethylbenzylammonium chloride and tributyl tetradecyl phosphonium chloride against a biofilm consortium. Although previously considered rare in nature, D-amino acids are now considered ubiquitous due to the advancement of analytical techniques and the increased interests in their biological functions. They are found in microorganisms, food, plants, animals, and even in humans (Konno et al., 2009). While the biological functions of D-amino acids are not fully understood, it is believed that they could serve as a signal molecule. Lam et al. (2009) stated that the synthesis of D-amino acids might be a common way of self-adjustment of cells to the changing environment. Kolodkin-Gal et al. (2010) found that D-methionine (D-met), D-tyrosine (D-tyr), D-leucine (D-leu), and D-tryptophan (D-trp) triggered the *Bacillus subtilis* biofilm’s disassembly. They also tested an equimolar mixture of four D-amino acids. Xu and Liu (2011) confirmed that 100 ppm (w/w) D-tyr triggered the biofilm dispersal in their test using activated sludge on membrane filters. Xu et al. (2012a, 2014) found that D-tyr and D-met were effective against the *D. vulgaris* biofilm on carbon steel coupons.

In this work, the efficacies of THPS combined with a mixture of D-amino acids against two oil-field biofilm consortia (labeled as Consortium I and Consortium II) were investigated. An equimolar mixture of four D-amino acids (D-met, D-tyr, D-leu, and D-trp) were tested with 50 ppm THPS. Individual D-met and D-tyr were also tested with THPS for comparison.

## Materials and Methods

### Bacterium, Nutrient Medium, Biofilm Growth Substratum, and Chemicals

In this work, two biofilm consortia collected from an oil field were used to investigate the efficacy of 50 ppm THPS in combination with D-amino acids. The consortia were collected from oilfield operations in the form of planktonic cells in liquids in glass jars. The liquid samples were used to inoculate the ATCC 1249 medium that is a modified Baar’s medium. L-Cysteine at 100 ppm was added to the medium as an oxygen scavenger to eliminate any possible oxygen ingress.

Coin-shaped C1018 (UNS G10180) carbon steel coupons were used to grow biofilms. The composition of C1018 was (wt%): C 0.14–0.20, Mn 0.60–0.90, P 0.04, S 0.05, Si 0.15–0.30, and Fe balance. For each coupon, only the 1.2 cm^2^ top surface was exposed to the culture medium, and the remainder was painted with Teflon. Coupons were polished with 180, 400, and 600 grit sandpapers, sequentially. They were cleaned with isopropanol and dried under UV light for 20 min.

D-Amino acids, THPS, and all chemicals used in the culture medium were purchased from Sigma–Aldrich (St Louis, MO, USA) or Fisher Scientific (Pittsburgh, PA, USA). In the 50 ppm equimolar D-amino acid mixture (D-mix), the concentrations of D-met and D-trp in the mixture were much less than the concentration of each D-amino acid which was needed to enhance 50 ppm THPS in the mitigation of the *D. vulgaris* biofilm on carbon steel (Xu et al., 2012a, 2014).

### Biofilm Prevention and Biofilm Removal Tests

In order to evaluate the efficacies of the cocktails of THPS + D-amino acid(s), both biofilm prevention and biofilm removal tests were carried out in the lab. Before the operation, the culture medium, anaerobic vials, pipettes, and tweezers were sterilized in an autoclave at 121 °C for 20 min before use. D-Amino acids were not autoclaved due to possible oxidation at a high temperature. Their stock solutions were filter-sterilized. The culture medium and solutions containing the biocide treatment chemicals were sparged with filtered N_2_ for 45 min to remove O_2_. In the biofilm prevention test, two duplicate coupons, 100 ml medium, treatment chemicals, and 1 ml biofilm consortium seed culture were put into each 125 ml anaerobic vial in an anaerobic chamber. The initial planktonic cell concentration right after inoculation was 10^6^ cells/ml. The anaerobic chamber was sparged with filtered N_2_ for 45 min to remove oxygen before use. After the vials were sealed, they were placed in a 37°C incubator. After 7-day incubation, coupons were taken out for further measurement and observation. In the biofilm removal test, biofilms were first grown on coupons without treatment chemicals for 3 days to achieve maturity. Coupons covered by the mature biofilm were taken out and put into a phosphate-buffered saline (PBS) solution (pH 7.4) with treatment chemicals in a Petri dish for 3 h in the anaerobic chamber at room temperature. Coupons were taken out for analysis afterwards. Each test was repeated twice and triplicate coupons were used for each test condition.

### Enumeration of Sessile Cells

The Biosan Sani-Check SRB test kit (Sani-Check^®^ Product #100, Biosan Laboratories, Warren, MI, USA) was used for the most probable number (MPN) cell counts. The biofilm consortium on the coupon surface was removed with the small brush that was a part of the test kit. The brush was then inserted into the test kit’s vial containing a solid SRB culture medium for incubation at 37°C. The time it took to show the black color (FeS) reflected the SRB cell concentration based on vendor’s calibration (Xu et al., 2012a,b, 2014).

### Scanning Electron Microscope (SEM) for Biofilm Observation

Scanning electron microscope observation with a Model JSM-6390 SEM (JEOL Ltd., Tokyo, Japan) was used to observe the biofilm morphology. The coupons for SEM observation of biofilms were first submerged in 4% (w/w) glutaraldehyde for 2 h and then dehydrated in 25, 50, 75, and 100% (v/v) isopropanol sequentially for 5 min total. And then, the biofilms were dehydrated in a critical point dryer using CO_2_. Finally, biofilms covered coupons were coated with palladium to provide conductivity (Xu et al., 2012a,b, 2014). It should be noted that SEM images should not be used for quantitative cell counting because of uneven distribution of sessile cells.

### Confocal Laser Scanning Microscopy (CLSM) for Biofilm Observation

Confocal laser scanning microscopy using Model LSM 510 microscope (Carl Zeiss, Jena, Germany) was used to detect living and dead cells in biofilms. The dyes used to stain the biofilms were in the L7012 Live/Dead^®^ BacLight^TM^ Bacterial Viability Kit (Life Technologies, Grand Island, NY, USA), in which SYTO 9 is a green-fluorescent stain and propidium iodide a red-fluorescent stain. Thus, living cells appear as green color at an excitation wavelength of 488 nm and dead cells red at 559 nm. Before being put into the dye solution for 15 min in a dark condition, coupons were washed with the PBS buffer for 15 s three times to wash away the culture medium, biocide residue, and planktonic cells (Mah et al., 2003; Turonova et al., 2015).

## Results

### Oil-Field Biofilm Consortia

**FIGURE 1:**
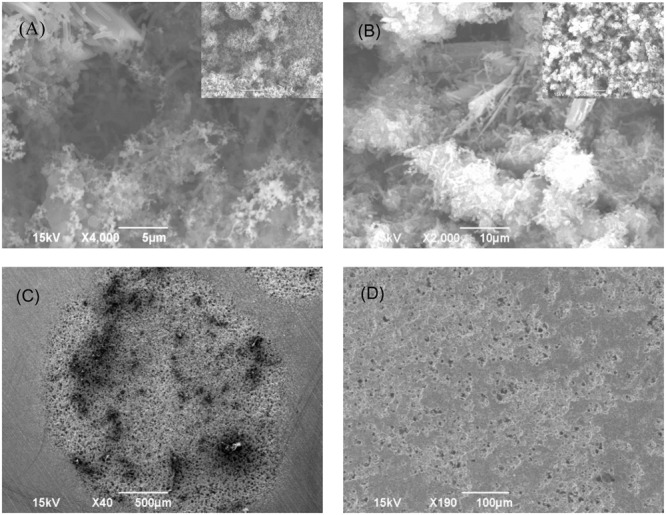
**Biofilm images and bare coupon surface images after biofilm removal for coupons after 10-day incubation (without treatment chemicals) at 37°C: (A) Consortium I biofilm, (B) Consortium II biofilm, (C) coupon surface after removal of Consortium I biofilm, and (D) coupon surface after removal of Consortium II biofilm**.

Biofilm Consortia I and II were supplied by an oil and gas company. **Figure 1** shows that both biofilm consortia formed robust biofilms on coupons after incubation in the culture medium for 10 days at 37°C. The images also reveal that the sessile cells in both biofilm consortia appeared to have different morphologies, indicating that they were mixed-culture consortia. Since the ATCC 1249 culture medium is designed for SRB, the dominant sessile population observed was likely SRB. After the biofilms were removed, pits underneath the biofilms resembled characteristic MIC pits (**Figure 1**). The specific weight losses of the coupons were 0.0047 and 0.0058 g/cm^2^ for Consortium I and Consortium II, respectively. Both values were much larger than the 0.0018 g/cm^2^ caused by pure-strain *D. vulgaris* incubated under the same conditions.

The phylogenetic identification of Consortia I and II shown in **Table 1** was carried out by Ecolyse, Inc. (College Station, TX, USA). Ecolyzed used the bacterial tag-encoded FLX amplicon pyrosequencing (bTEFAP) method with 515F-GTGCCAGCMGCCGCGGTAA and 806R-TAATCTWTGGGVHCATCAGG as primers for DNA analysis. A forward primer and a reverse fusion primer were used for sample amplification. The amplified product was processed to create single-stranded DNA using the Ion PGM protocols by Life Technologies (Carlsbad, CA, USA). Sequencing was performed using the Ion PGM semiconductor sequencing method by Life Technologies.

**Table 1:** Metabolic assignments of dominant bacterial species (%).

Species	Consortium I	Consortium II	Trait
*Bacillus* sp.	<1	6.0	Biosurfactant producing; Varies
*Bacterioides* sp.	2.9	1.4	Fermenting bacteria
*Garciella* sp.	27.7	78.8	NRB; Sulfidogen; TRB
*Salmonella enterica*	61.5	0	SRB; TRB
*Soehngenia* sp.	4.1	0	Fermenting bacteria
*Tepidibacter* sp.	2.7	13.6	Biodeg (HC)

In **Table 1**, “Sulfidogens” are bacteria that reduce sulfur and produce sulfide. “Biodeg,” abbreviated from biodegradation, refers to bacteria utilizing substrates or compounds that cannot be used by most of the other bacteria. “HC” means hydrocarbon. The metagenomics data reveal that *Bacillus* sp., *Bacterioides* sp., *Garciella* sp., *Salmonella enterica*, *Soehngenia* sp., and *Tepidibacter* sp. were detected in Consortia I. Consortium II contained all these species, except *Salmonella enterica* and *Soehngenia* sp. The MIC caused by *Bacillus* sp. were studied since 1990s (Jack et al., 1992). *Bacteroides* sp. are normally found in mammals (Huang et al., 2011). *Bacteroides* sp. secret organic acids which are able to cause Type II MIC (Zhu et al., 2003). *Garciella* sp. and *Soehngenia* sp. were collected from the oil and gas field (Miranda-Tello et al., 2003; Kano et al., 2009; Naranjo-Briceño et al., 2012). *Garciella* sp. may cause MIC by producing hydrogen sulfide from the thiosulfate reduction (Miranda-Tello et al., 2003). *Salmonella* sp. and *Soehngenia* sp. are related to MIC because they produce hydrogen sulfide in their metabolisms (Barrett and Clark, 1987; Parshina et al., 2003). *Tepidibacter* sp. were found in oil fields (Tan et al., 2012).

### Individual D-Amino Acids as Biocide Enhancers in the Mitigation of Field Biofilm Consortia

In our previous tests, the cocktails of 50 ppm THPS + 100 ppm D-met and 50 ppm THPS + 1 ppm D-tyr both achieved 5 log reduction of the SRB sessile cell count against *D. vulgaris* on carbon steel coupons (Xu et al., 2012a, 2014). However, the data in **Table 2** illustrate that in the biofilm prevention tests against Consortia I and II, the same cocktails only achieved 1 or 2 log reduction of the SRB sessile cell count, much less than 5 for *D. vulgaris*. The data presented in **Table 2** also show that increasing D-tyr from 1 to 10 ppm did not improve the outcome. All these data suggest that the field biofilm consortia were much more recalcitrant than the pure-strain *D. vulgaris*.

**Table 2:** SRB sessile cell counts of Consortia I and II after 7-day biofilm prevention test using D-tyr and D-met.

Biofilm	Treatment	MPN sessile cell count (cells/cm^2^)
Consortium I	No treatment chemical (control)	10^7^
	50 ppm THPS	10^7^
	50 ppm THPS + 100 ppm D-met	10^6^
	50 ppm THPS + 1 ppm D-tyr	10^5^
	50 ppm THPS + 10 ppm D-tyr	10^5^

Consortium II	No treatment chemical (control)	10^7^
	50 ppm THPS	10^7^
	50 ppm THPS + 100 ppm D-met	10^6^
	50 ppm THPS + 1 ppm D-tyr	10^6^
	50 ppm THPS + 10 ppm D-tyr	10^6^

### D-Amino Acid Mixture as Biocide Enhancer Against Consortium I

For the mitigation of the biofilm Consortium I, **Table 3** data reveal that 50 ppm D-mix enhanced 50 ppm THPS in both biofilm prevention and biofilm removal tests. In the biofilm prevention test, 50 ppm THPS without enhancement did not reduce the SRB sessile cell count on the coupon surface compared with the control without treatment. While the treatment with 500 ppm D-mix without THPS achieved 2 log reduction of the SRB sessile cell count. The data in **Table 3** show that the combination of 50 ppm THPS + 50 ppm D-mix was able to achieve 4 log reduction of the SRB sessile cell count. This demonstrated that the 50 ppm D-mix enhanced 50 ppm THPS in the biofilm prevention test for biofilm Consortium I considerably.

**Table 3:** SRB sessile cell counts of Consortium I biofilm after 7-day biofilm prevention test using a D-amino acid mixture.

Treatment	MPN sessile cell count (cells/cm^2^)
No treatment chemical (control)	10^7^
50 ppm THPS	10^7^
50–500 ppm D-mix	10^5^
50 ppm THPS + 50 ppm D-mix	10^3^

**FIGURE 2:**
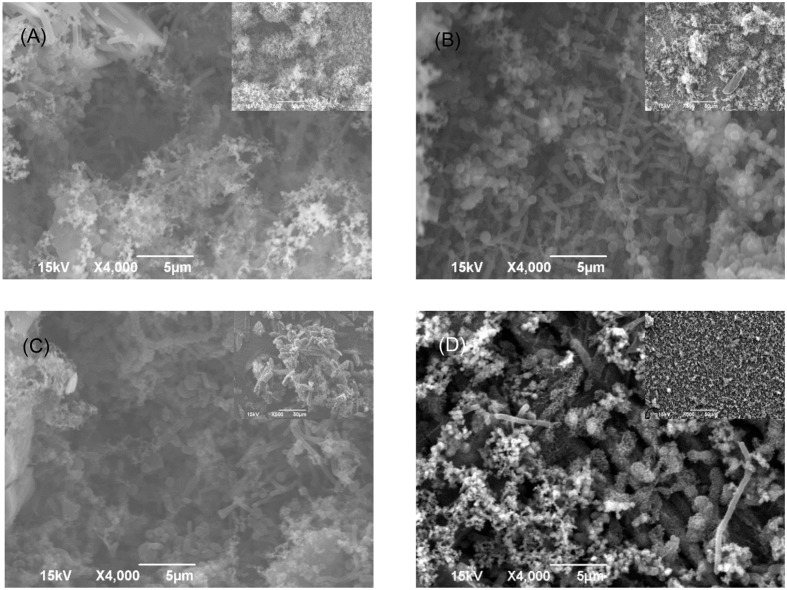
**SEM images of Consortium I biofilm after 7-day incubation in the biofilm prevention test with: (A) no treatment chemical (control), (B) 50 ppm THPS, (C) 50 ppm D-mix, and (D) 50 ppm THPS + 50 ppm D-mix**. The scale bar in the inserted small image is 50 μm.

**FIGURE 3:**
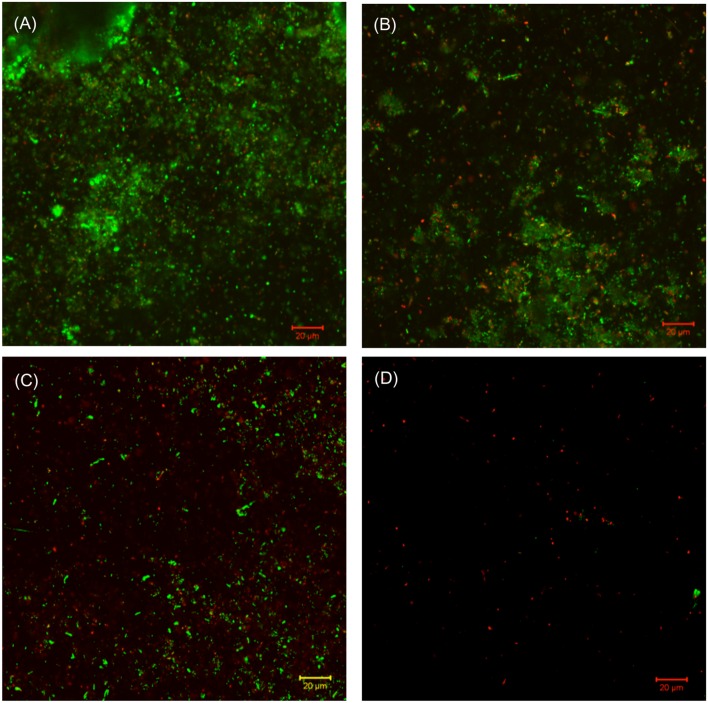
**CLSM images of biofilms after 7-day incubation in the biofilm prevention test with: (A) no treatment chemical (control), (B) 50 ppm THPS, (C) 50 D-mix, and (D) 50 ppm THPS + 50 ppm D-mix**.

The SEM images of the biofilm Consortium I after a 7-day biofilm prevention test in **Figure 2** are consistent with the results in **Table 3**. Different cell shapes in the biofilm image confirmed that this was a mixed-culture biofilm. Sessile cells were easily found on coupons treated with either 50 ppm THPS or 500 ppm D-mix alone. However, with the 50 ppm THPS + 50 ppm D-mix cocktail treatment, the amount of sessile cells was much less than the amount of sessile cells after treatment with either 50 ppm THPS or 500 ppm D-mix. The CLSM images support the cell count data and are consistent with the SEM images. On the control coupon surface in **Figure 3A**, abundant living cells can be seen. In the treatments of 50 ppm THPS alone and 50 ppm D-mix alone in **Figures 3B,C**, dead cells as are seen but living cells are far more abundant. In **Figure 3D**, the amounts of both living and dead cells are much less and most cells were dead cells. It should be noted that although sessile cells were found on the SEM image in **Figure 2D**, they were dead cells that had not yet detached from the coupon according to the CLSM image in **Figure 3D**.

**FIGURE 4:**
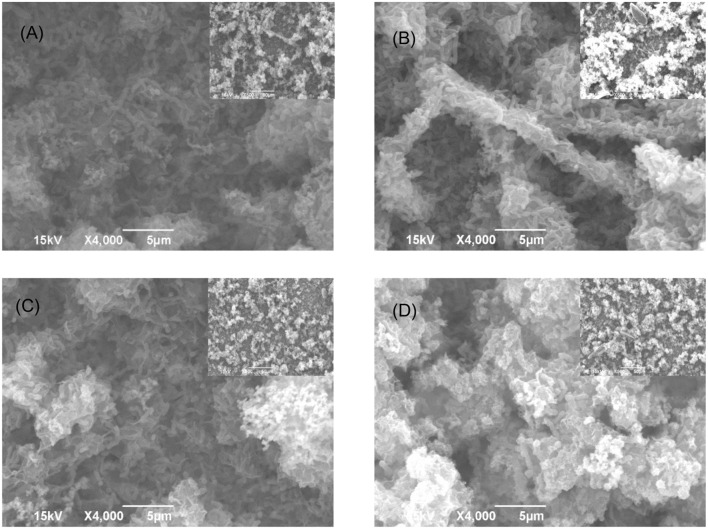
**SEM images of Consortium I biofilm after 3-h treatment in PBS buffer containing: (A) no treatment chemical (control), (B) 50 ppm THPS, (C) 100 ppm D-mix, and (D) 50 ppm THPS + 50 ppm D-mix, in the biofilm removal test**. The scale bar in the inserted small images is 50 μm.

**FIGURE 5:**
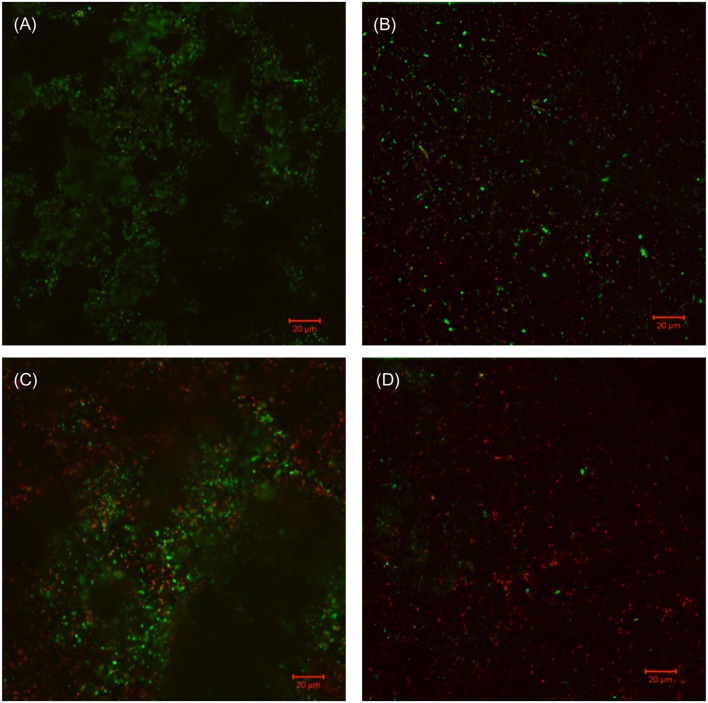
**CLSM images of biofilms in the 3-h treatment in PBS buffer with: (A) no treatment chemical (control), (B) 50 ppm THPS, (C) 50 ppm D-mix, and (D) 50 ppm THPS + 50 ppm D-mix**.

Similar efficacies were obtained in the biofilm removal test for biofilm Consortium I. The data provided in **Table 4** illustrate that the separate applications of 50 ppm THPS and 500 ppm D-mix had limited effects on Consortium I. It was found that 50 ppm THPS achieved no reduction of the SRB sessile cell count and 500 ppm D-mix achieved only 2 log reduction of the SRB sessile cell count. While the cocktail of 50 ppm THPS + 50 ppm D-mix provided a 4 log reduction of the SRB sessile cell count after the 3-h biofilm removal test and the SEM images support the reduction of the sessile cells (**Figure 4**). **Figure 4** shows that sessile cells were present in the biofilm of Consortium I after treatment with either 50 ppm THPS or 100 ppm D-mix alone. The same trend was observed in the CLSM images (**Figure 5**) showing that more living cells were found on the control coupon surface than on the coupon surfaces treated with 50 ppm THPS and 50 ppm D-mix alone. On the coupon surface treated with 50 ppm THPS + 50 ppm D-mix, more dead cells and less living cells were found than on the coupon surface in the other three treatments.

**Table 4:** SRB sessile cell counts of Consortium I after 3-h biofilm removal test.

Treatment	MPN sessile cell count (cells/cm^2^)
No treatment chemical (control)	10^7^
50 ppm THPS	10^7^
50–500 ppm D-mix	10^5^
50 ppm THPS + 50 ppm D-mix	10^3^

### D-Amino Acid Mixture as Biocide Enhancer Against Consortium II

**FIGURE 6:**
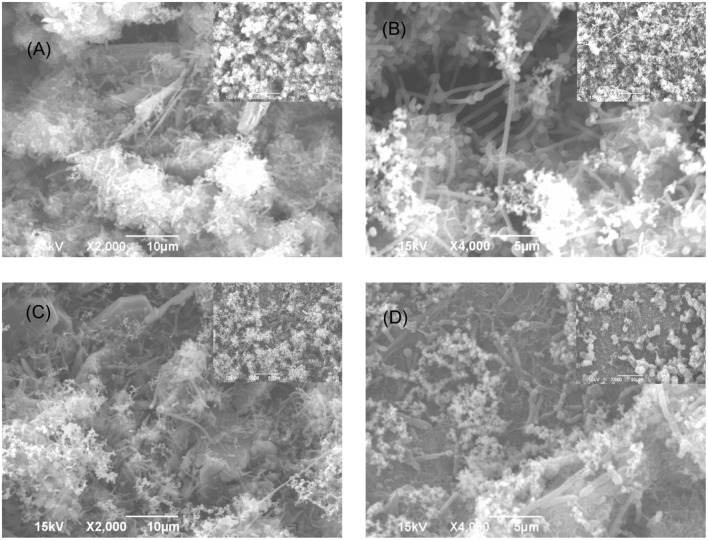
**SEM images of Consortium II biofilm after 7-day incubation in the biofilm prevention test with: (A) no treatment chemical (control), (B) 50 ppm THPS, (C) 100 ppm D-mix, and (D) 50 ppm THPS + 50 ppm D-mix in the biofilm prevention test**. The scale bar in the inserted small images is 50 μm.

**FIGURE 7:**
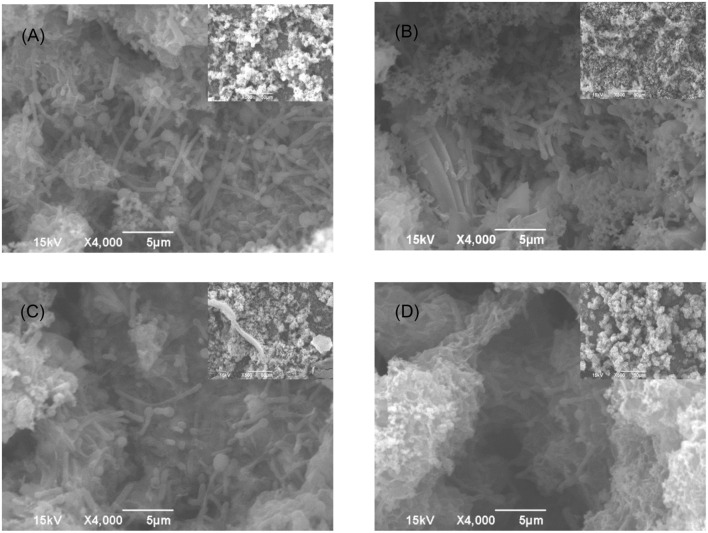
**SEM images of Consortium II biofilm after 3-h treatment in PBS buffer containing: (A) no treatment chemical (control), (B) 50 ppm THPS, (C) 100 ppm D-mix, and (D) 50 ppm THPS + 50 ppm D-mix in biofilm removal test**. The scale bar in the inserted small images is 50 μm.

**FIGURE 8:**
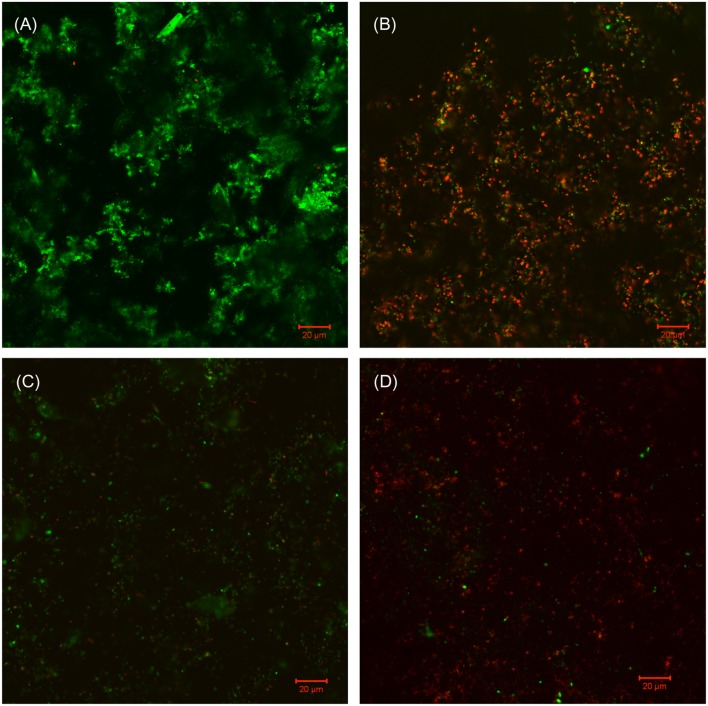
**CLSM images of Consortium II biofilm after 7-day incubation in the biofilm prevention test with: (A) no treatment chemical (control), (B) 50 ppm THPS, (C) 50 ppm D-mix, and (D) 50 ppm THPS + 50 ppm D-mix in the biofilm prevention test**.

**FIGURE 9:**
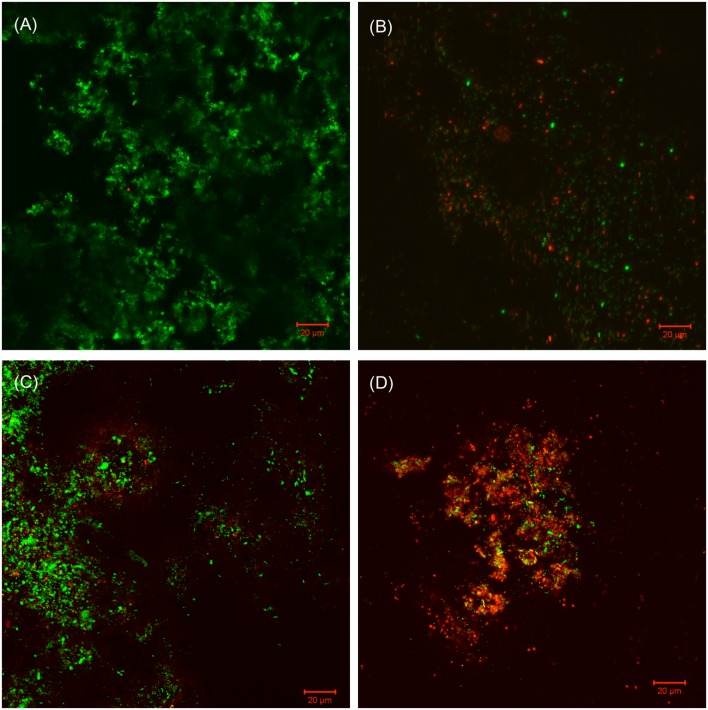
**CLSM images of Consortium II biofilm after 3-h treatment in PBS buffer containing: (A) no treatment chemical (control), (B) 50 ppm THPS, (C) 50 ppm D-mix, and (D) 50 ppm THPS + 50 ppm D-mix in the biofilm removal test**.

Similar treatment outcomes were obtained for Consortium II. It was found that 50 ppm D-mix enhanced 50 ppm THPS in both biofilm prevention and biofilm removal tests. Comparable to the mitigation of Consortium I, 50 ppm THPS alone had no effect on the SRB sessile cell count in both biofilm prevention and biofilm removal tests for Consortium II compared with the untreated control (**Tables 5** and **6**). The cocktail of 50 ppm THPS + 50 ppm D-mix achieved 3 log reduction of the SRB sessile cell count in both biofilm prevention and biofilm removal tests for biofilm Consortium II. In **Figures 6** and **7**, sessile cells are easily seen on the following: the control coupon (no treatment), the coupon treated with 50 ppm THPS, and the coupon treated with 500 ppm D-mix. Although sessile cells are still noticeable on the surface of coupons treated with 50 ppm THPS + 50 ppm D-mix, they are less abundant. Moreover, according to the CLSM images in **Figures 8** and **9**, on the coupon surfaces treated with 50 ppm THPS + 50 ppm D-mix (**Figures 8D and 9D**), most cells were dead cells. The data suggest that the binary combination of 50 ppm THPS and 50 ppm D-mix had an excellent efficacy against Consortium II owing to the enhancement of the D-mix.

**Table 5:** SRB sessile cell counts of Consortium II after 7-day biofilm prevention test.

Treatment	MPN sessile cell count (cells/cm^2^)
No treatment chemical (control)	10^7^
50 ppm THPS	10^7^
50–500 ppm D-mix	10^6^
50 ppm THPS + 50 ppm D-mix	10^4^

**Table 6:** SRB sessile cell counts of Consortium II after 3-h biofilm removal test.

Treatment	MPN sessile cell count (cells/cm^2^)
No treatment chemical (control)	10^7^
50 ppm THPS	10^7^
50–500 ppm D-mix	10^6^
50 ppm THPS + 50 ppm D-mix	10^4^

## Discussion

The exact mechanism of how D-amino acids enhance biofilm mitigation is not completely understood. It was hypothesized that D-amino acids trigger biofilm disassembly by replacing the D-alanine terminus in peptidoglycans that exist in all bacterial cell walls (Kolodkin-Gal et al., 2010). Kolodkin-Gal et al. (2010) and Xu et al. (2014) found that adding a high concentration of D-alanine (D-ala) rendered D-met ineffective. It was also suggested that D-amino acids may modify the synthesis of peptidoglycans (Lam et al., 2009). Cava et al. (2011) suggested that D-amino acids were necessary in the remodeling of the cell wall structure. Leiman et al. (2013) pointed out that D-tyr, D-leu, and D-trp inhibited the formation of the *B. subtilis* biofilm because these D-amino acids interfered with protein synthesis. Their data suggested that D-tyr acted as a growth inhibitor toward *B. subtilis.* It is highly possible that specific D-amino acids are effective for specific bacteria. Sarkar and Pires (2015) tested millimolar concentrations of several D-amino acids against some bacteria and found no inhibition of biofilm formation. This is consistent with the view proposed in Xu et al.’s (2012a,b, 2014) work that a biocide stress is needed.

It is not a surprise that the combination of THPS and D-amino acid mixtures showed a synergetic effect in the mitigation of field biofilm consortia. The synergy between biocides and other agents, such as the injection of nitrate, has been demonstrated in the mitigation of reservoir souring (Xue and Voordouw, 2015). The results above proved the hypothesis that a D-amino acid mixture instead of an individual D-amino acid would be needed in the mitigation of a biofilm consortium. The cocktail of 50 ppm THPS + 50 ppm D-mix achieved a better efficacy (1 log more reduction) for Consortium I than for Consortium II, suggesting that biofilm Consortium II might be more recalcitrant. It was also possible that a different D-amino acid mixture may work better for Consortium II. Different microbial species in the biofilm consortia probably required different D-amino acids as the biofilm dispersal factor. It is reasonable that in the mitigation of other biofilm consortia, the particular D-amino acid mixture in this work may need adjustments. Optimization is likely needed to decide a cost-efficient D-amino acid mixture that offers a good efficacy.

The comparison of SEM and CLSM biofilm images in this work suggests that SEM images could give a false impression that sessile cells were abundant while in reality the cells might be mostly dead cells. This should not be a problem if a biofilm is already detached from a coupon surface. Unlike CLSM, SEM can be used to show different cell shapes in a mixed-culture biofilm.

Hence, this lab work showed that 100 ppm D-met and 1 ppm D-tyr individually did not enhance or only slightly enhanced 50 ppm THPS in the mitigation of two field biofilm consortia on C1018 coupon surfaces although they worked very well for the pure-strain *D. vulgaris* biofilm. This work demonstrated that an equimolar mixture at a total concentration of 50 ppm consisting of D-met, D-tyr, D-leu, and D-trp greatly enhanced 50 ppm THPS in the mitigation of the biofilm consortia. It was found that the cocktail achieved extra 4 log reduction of the SRB sessile cell counts in both the biofilm prevention and biofilm removal tests for biofilm Consortium I compared with the treatment with 50 ppm THPS alone, while for Consortium II it was extra 3 log.

## Author Contributions

YL, RJ, and HA-M designed the experiments and conducted the data analysis. YL, DX, and TG wrote and edited the manuscript. All authors participated in discussion about the results and the manuscript.

## Conflict of Interest Statement

The authors declare that the research was conducted in the absence of any commercial or financial relationships that could be construed as a potential conflict of interest.
